# The information and support needs of patients living with inflammatory bowel disease: A qualitative study

**DOI:** 10.1177/1742395320968617

**Published:** 2020-10-26

**Authors:** Paige Karadag, Beth Morris, Kerry Woolfall

**Affiliations:** 1 School of Psychology, University of Liverpool, Liverpool, UK; 2 Department of Public Health, Policy and Systems, University of Liverpool, Liverpool, UK

**Keywords:** Inflammatory bowel disease, qualitative, social media, quality of life, information

## Abstract

**Objectives:**

To explore patients’ experiences of living with inflammatory bowel disease (IBD) with a focus on their information and support needs.

**Methods:**

Qualitative interview study involving adults diagnosed with IBD recruited through social media. Interviews were audio recorded, transcribed and data were analysed thematically.

**Results:**

Interviews with 15 patients (9 females, 6 males) highlighted how misdiagnosis or hesitation to diagnose had caused frustration and anxiety. Once diagnosed, only a few participants received detailed information about IBD from their doctor. Negative experiences shared on social media caused initial anxiety, as individuals assumed that they may have a similar experience, yet online communities enabled insights into the experiences of others, helping patients adjust to living with IBD. Participants described both positive and negative impacts of living with IBD, including improved confidence and periods of anxiety.

**Discussion:** Our findings highlight the importance of clear information and support from health professionals, as well as the benefits of online communities for ongoing support. At the point of diagnosis, patients would benefit from information about what IBD is, as well as how it may impact day to day life from doctors so that social media is not the only source of initial information about IBD.

## Introduction

Inflammatory bowel disease is a chronic, incurable, autoimmune disease occurring in mainly western and urbanised areas.^1^ The two main forms of IBD are Crohn’s Disease (CD) and Ulcerative Colitis (UC). IBD has a global prevalence of 0.3%, affecting^2^ over 300,000 in the UK alone.^3^ The National IBD audit estimated annual treatment and support costs to the NHS to be over £1 billion.^4^

IBD has a considerable impact on an individual’s daily life. Bowel related symptoms such as urgency, incontinence, frequency, diarrhoea and passing blood resulted in lifestyle changes, dietary restrictions and the need to maintain close proximity to a toilet.^5,6^ Embarrassment and stigma of IBD symptoms, fear of negative long-term effects, and unpredictability of flares, have been shown to have negative psychological impacts,^7^ including apprehension about loss of control and negative body image. IBD populations are twice as likely to experience generalised anxiety disorder^8^ and report significantly higher levels of depression compared to non IBD populations.^9^ Living with IBD also negatively impacts family life, with between one fifth and one third of IBD patients reviewed selecting voluntary childlessness due to concerns about pregnancy or how IBD would affect parenting ability.^10^

The few studies that have explored patient perspectives on clinical healthcare services for IBD largely indicate a dissatisfaction with care. Patients report feeling abandoned by healthcare professionals, who only treat clinical symptoms rather than the impact of IBD in its entirety on an individual’s life.^11^ A survey by Baars et al.,^12^ found that more than half of IBD patients surveyed want to be better informed about their disease, specifically regarding new medication options. In 2011, Bernstein et al. reported patients' dissatisfaction with information provided at diagnosis. Consequently, it is important that patients' information needs are better understood to inform healthcare professionals on how they can improve the quality and content of information they provide.

Despite the profound impact IBD has upon an individual’s quality of life and life choices, there is a lack of detailed insight into what patients view as the barriers and facilitators to support from healthcare professionals, as well as wider social and family networks. This study aimed to explore patients’ experiences of living with inflammatory bowel disease (IBD) with a particular focus on their medical information and emotional support needs to help inform future healthcare practice.

## Materials and methods

We designed a qualitative interview study informed by a phenomenological approach^11^ to explore patients’ experiences of living with IBD in a flexible way that is appropriate to a potentially emotive topic.^13,14^

### Recruitment

Participants were recruited online via social media such as Facebook, Twitter and Instagram. The advert was posted on social media platforms, which included details of how individuals could contact the study team to register their interest in taking part. Purposive sampling was also used by the researchers (PK, female psychologist BSc (Hons) and BM, female psychologist BSc (Hons)), who contacted the Instagram accounts of IBD support groups, and individuals who use the platform to share their experiences of living with IBD.

### Inclusion criteria

Participants were eligible to participate if they had a diagnosis of CD or UC, lived in the UK, were aged between 18 and 40 (to reflect age of onset of IBD^15,16^) and spoke English. The age limit enabled the inclusion of individuals diagnosed during childhood or adolescence to discuss past experiences. If all eligibility criteria were met, individuals were sent a copy of the participant information sheet detailing the study aims, reasons for conducting the study, and what interview participation would involve, which they were asked to read before the scheduled interview date.

### Exclusion criteria

Individuals who did not speak English, lived outside of the United Kingdom or had a personal relationship with the researcher.

### Data collection

Data were collected through audio recorded semi- structured interviews conducted by PK and BM. Participants could choose from either telephone or face to face interviews at the University of Liverpool. A topic guide was developed using existing literature,^5,12,17,18^ and aimed to explore patient experiences of living with IBD with a focus on knowledge and information needs, particularly around the point of diagnosis, early symptoms and diagnosis, treatments and concerns, barriers and facilitators to support, and impact on family functioning and lifestyle (see Table 1).

**Table 1.** Topics covered in the interviews and examples of questions asked.

Topic covered	Example questions
Impacts of early symptoms and the diagnosis process	– How long did it the process take from first symptoms to diagnosis? – Could you tell me a bit more about your experience of the diagnosis process? Prompt: *Explore length of time, number of doctors visited, and referrals?* – How did you feel when you were initially diagnosed with IBD?
Clarity of knowledge regarding IBD and IBD treatments	– How much knowledge of IBD did you have before diagnosis? – Following diagnosis, did you gain knowledge about IBD? If yes, how did you go about it? *Prompts: how easy it was to access information where that information was from, is there anything that could be changed to help people access information?*
The impact IBD has on quality of life including family and social functioning.	– What impact does IBD have upon your life? – Does IBD impact upon your social life? If yes, could you tell me a bit more about that? – Does IBD impact upon your family life? If yes, could you tell me a bit more about that?
Challenges and support	– Are there any challenges you have faced in relation to you IBD? – How would you describe the quality of care you have received from the medical professionals you have met? – Do you get any support from family or friends? Can you tell me more about this and what you find useful?

Based on previous qualitative studies,^19,20^ we anticipated interviewing 14–25 participants. PK and BM responded to individual requests to participate in sequential order, confirmed eligibility and emailed them a participant information sheet. Audio-recorded verbal consent was sought before the interview began. Digital audio recordings were transcribed verbatim, anonymised and checked for accuracy. Respondent validation was used to add unanticipated topics to the topic guide as interviewing and analysis progressed.^21^ PK and BM made field notes after interviews. Interviews aimed for data saturation, that is, the point where no new major themes are discovered in analysis.^22,23^ Screening stopped when data saturation was reached. Audio files were transcribed verbatim, anonymised and checked for accuracy. Accuracy was checked by PK and BM listening to the audio files whilst reading the transcripts following the initial transcription, to check the audio data matched the written data, and that all identifying information was removed. Alongside removing identifying information, pseudonyms were used to guarantee anonymity of participants.

### Data analysis

PK and BM (psychologists) led the analysis with assistance from KW (female social scientist PhD) who second coded 10% of transcripts. In line with a phenomenological approach which underpinned the study,^11^ thematic analysis was interpretative and iterative, aiming to identify patterns of meaning within the dataset regarding individual’s experiences of living with IBD.^13^ Our approach to thematic analysis was reflexive and recursive, in line with Braun and Clarke’s six phase approach.^24^ As detailed in Table 2, regular meetings were held to discuss transcripts and develop the coding framework. NVivo V.12 software^25^ was used to assist with data organisation and add rigour to the process by enabling clear coding structures. Interview quotations were presented with pseudonyms to demonstrate the identified themes. Where quotations have been shortened for brevity or to remove identifiable information, omitted text has been marked with “…” and explanatory text is in brackets.

**Table 2.** Approach to data analysis.

Phase	Description
1. Familiarization	PK and BM read and re-read interview transcripts and their notes from interviews, noting down initial ideas on potential themes and connections between participant accounts.
2. Generating initial codes	In line with a phenomenological approach PK, BM and KW initially discussed data-driven codes and concepts that reflected participant’s subjective experiences of living with IBD, including their support needs. Broad themes were identified to develop an initial coding framework. Analysis was based on thematic analysis, a method for identifying, analysing and reporting patterns (or themes) within data.
3. Constructing themes	Meaning patterns were noted and mapped. PK and BM initially focussed constructing themes for two transcripts each and shared the initial themes/potential coding frameworks with KW. KW second coded the four transcripts using the initial coding frames and made notes on any new themes identified and how the framework could be refined. Regular meetings were held to discuss and develop the coding framework.
4. Revising and defining themes	Following review and reconciliation by PK and BM, revised coding frames were subsequently developed and ordered into themes and sub themes (nodes) within the *NVivo* Database.
5. Completion of coding of transcripts	PK and BM completed coding transcripts and developing the framework and further refining themes deemed appropriate, reflecting on the study aims and theoretical framework.
6. Write up	PK, BM and KW developed the initial manuscript. KW led the final development of themes reflecting on the connections with existing literature during the write-up phase in collaboration with PK and BM.

**Table 3.** Table of themes and subthemes.

Theme	Sub Themes
Misdiagnosis and hesitation caused frustration and prolonged suffering.	• Length of diagnosis process. • Misunderstanding and misdiagnosis. • Frustration and upset.
Information needs at the point of diagnosis.	• Lack of knowledge of IBD prior to diagnosis. • Doctors provided limited information regarding IBD at diagnosis.
Access to specialist staff facilitated trust and a change for the better.	• Dismissal from General Practitioners. • High quality of care and support from IBD nurses and consultant gastroenterologists.
The positive and negative impacts of IBD upon individual wellbeing.	• Stress, fear and depressed mood as a result of IBD. • Showing resilience in spite of IBD challenges.
Family and partners as a source of emotional and practical support.	• Mothers looked after individuals during flare-ups. • Families research IBD to understand it better.
Sharing experiences and reducing stigma-the benefits of social media communities.	• Social media raising awareness of stoma bags. • Reassurance. • Better understanding of own health through seeing real life experiences of IBD online.

### Ethical considerations

Ethical approval was granted by The University of Liverpool research ethics committee (Reference number 3903). The interviewer explained participant confidentiality and anonymity and read out the consent form. Participants indicated consent verbally over the telephone. A copy of the consent form was later sent to the participant via email. Audio recordings and interview transcripts were stored securely in a shared team drive on a secure university computer.

## Results

### Participants

**Figure 1.**
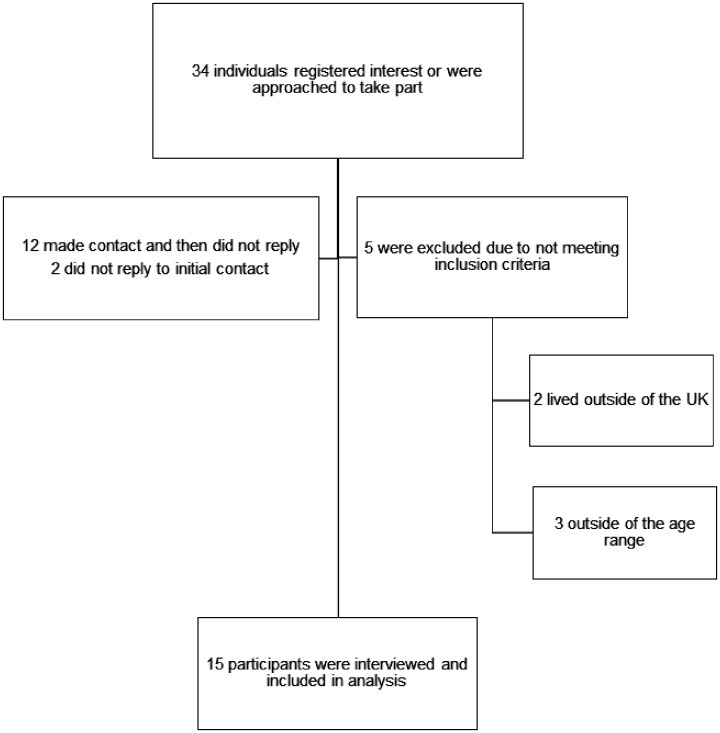
Recruitment flow chart.

Data saturation was reached at 15 participants, including 9 females and 6 males (aged 20–40, mean 28 years) (See Figure 1). Participants lived across the UK, seven had a diagnosis of CD and eight had a diagnosis of UC. Interviews averaged at 33 minutes in length (ranging from 19 to 71 minutes). All interviews were conducted over the phone from a private office on the University of Liverpool campus, without any other persons present.

### Results

Six themes were determined from analysis including: 1) *Misdiagnosis and hesitation caused frustration and prolonged suffering*, which describes the reduction in quality of life experienced when treatment was withheld, 2) *information needs at the point of diagnosis*, outlines lack of IBD knowledge prior to diagnosis, a general absence of information distributed by HCPs, and the subsequent need for individuals to seek information for themselves. 3) *Access to specialist staff facilitated trust and a change for the better*, encompasses how participants generally perceived improved quality of care from specialists, and the importance of IBD nurses 4) *positive and negative impacts of IBD upon individual wellbeing*, includes how participants felt they had learnt new things since living with IBD, 5) *Family and partners as a source of emotional and practical support*, highlights the important role of families in providing support and concerns about how IBD disrupts family life, and 6) *Sharing experiences and reducing stigma- the benefits of social media communities*, how shared experiences provided comfort for participants, and how this frequently occurs via social media platforms (See Table 3).

### Misdiagnosis and hesitation caused frustration and prolonged suffering

The majority of participants described how they were either misdiagnosed or medical professionals hesitated to make a diagnosis, leaving symptoms untreated for as long as five years. *‘I felt left to my own devices for quite a while’* (Katie aged 32) and *‘I kept being written off by the doctors’* (Maddy aged 22). This resulted in frustration as individuals would never be able to get back the months or years where quality of life had been significantly reduced due to untreated symptoms. In some cases, doctors attributed weight loss and fatigue to work stress, depression or anorexia nervosa, leading patients to believe *“every time they looked at me they thought I was lying about my illness”* (Bobby, aged 39).

**Jack, aged 22:** *we had to push a lot for them to believe that it was like actually a physical kind of condition rather than just a mental one.*

During interviews participants described their relief upon receiving their IBD diagnosis, as they would now be taken seriously by medical professionals and would receive appropriate treatment.

### Information needs at the point of diagnosis

Many participants had not heard of IBD prior to diagnosis, yet only two stated that their doctor had provided them with written information about the disease. An information leaflet about IBD was deemed the least amount of information that should be provided at the point of diagnosis.

**Steve, aged 40:** *I don’t think I was given a lot of information.*

**Katie, aged 32:** *I think maybe at least being given a leaflet or something to take away when you’re in hospital would be massively helpful.*

However, information about IBD had been *“easy”* (Maddy aged 22) to find through internet searches and appeared to have helped people gain an understanding of how IBD was likely to impact their lives.

**Sally, aged 27:** *I don’t feel like I will have got any of that directly from the NHS… it’s more about you know real-life – how it genuinely affects people.*

Nevertheless, some stated that they felt overwhelmed by the amount and content of online information, and cautioned that not all information available online was positive, and it was often unfiltered. Some of the online content had caused panic and anxiety, as many assumed the negative experiences they read about would happen to them.

**Katie, aged 32:** *I only did one google search and then it started to scare me… your mind takes over about all of the things that can go wrong.*

One participant recalled viewing a post on a Facebook group shortly after diagnosis, which gave her an “*end of the world situation in my head”* (Chloe, aged 25). However, eight years on she reflected that her own personal experience *“wasn’t half as bad”* (Chloe, aged 25). Many stated how they would have preferred written information from doctors at the point of diagnosis rather than relying on online materials.

### Access to specialist staff facilitated trust and a change for the better

Some patients described their diagnosis as a point at which to *“get my life back and start doing what I want to do”* (Gareth, aged 24). Following referral to consultants, specialist IBD nurses or surgeons, individuals’ experiences appeared to positively change. The quality of care provided by consultants was emphasised, with one participant explaining how their consultant *“changed my life for the better”* (Steve, aged 40). Good quality and open communication about IBD helped make them feel “*incredibly safe … [and I] started rebuilding my trust again with the NHS”* (Gareth, aged 24).

IBD nurses were highly valued. Participants reported *‘I’ve got a very very good IBD nurse’* (Kirsty aged 35) and *‘my IBD nurses, that team are incredible’* (Katie aged 32). Participants appreciated having easy access to nursing expertise with direct phone numbers that could be used to contact the nurses with queries. This resource helped address issues with prompt advice over the phone, or by arranging clinic appointments.

**Noah, aged 25:** *they were only kind of a phone call away…I’d phone them on the Monday and they’d have me in clinic on the Thursday.*

**Sally, aged 27:** *I think definitely she’s been like an integral part of like my mind set now as well, and like my road to recovery cause I just feel like she’s just sorted it all for me*

### The positive and negative impacts of IBD upon individual wellbeing

When reflecting on life with IBD, nearly all participants provided examples of both positive and negative impacts on their wellbeing. The majority of these were related to individuals’ mental health or personal growth.

Despite facing numerous challenges, many described how they had developed a new-found confidence and determination. For example, participants recognised that living with IBD *“does show how resilient you can be”* (Kirsty, aged 35).

**Gareth, aged 24:** *I never felt emotionally low. If anything, I felt empowered.*

**Sally, aged 27:** *while you know your body is not going to be exactly how it was before and you’re maybe not going to be able to do everything you did before, it’s more like a determination to say well what can I do?*

Others described having *‘more like knowledge of disabled people’* (Katie aged 32), whilst one individual believed that without IBD he *‘wouldn’t be the same person’* (Jack aged 22). Many stated that IBD had changed their perspectives on health, including new knowledge on how to eat a healthy diet and how to maintain a positive attitude in the face of adversity.

**Kirsty aged 35:** *learning to control diet … I’m probably heathier now in some respects than I ever was before I had Crohns*

**Steve, aged 40:** *The best advice that I ever had from my consultant… your bowel is a mirror of your mind, he said if you think yourself ill you’ll be ill*

However, thirteen participants also reported a “*major impact on my mental health*” (Bobby, aged 39), as a result of living with IBD. Several individuals described elevated stress levels stemming from aspects of their condition which they perceived were beyond their control and therefore acted as a source of anxiety, *“I got sort of like anxiety about leaving the house”* (Sally, aged 27). One interviewee stated that the biggest challenge of IBD was *‘how I feel about myself’* (Maddy, aged 22) and another reported a *“confidence issue”* (Stella, aged 21), following surgery to create a stoma. Most stated that these episodes were often short lived, as although *“it does bring you down you know sometimes”* (Noah, aged 25), those occasions mostly occur during flares. Despite this, Katie, aged 32 stated that she had *“never been offered counselling”* and argued that *“there should be things in place”* for mental distress.

**Fearne, aged 38:** *When I was feeling ill pretty much every day it was always on your mind and I think that can have a draining effect on you.*

A common fear amongst individuals was their health in the future, as flares could occur at any point with no current hope of a cure.

**Maddy, aged 22:** *just constantly praying that I’m not going to have anything bad happen.*

**Kirsty, aged 35:** *one of the hardest things to deal with, it’s not kind of knowing.*

### Family and partners as a source of emotional and practical support

Participants highlighted the importance of actions of supportive family members or partners for providing emotional support during time of illness, as well as practical support such as accompanying them to appointments and helping to administer injections. As the following quote illustrates, partners and family members provided much needed encouragement, helping to reduce fears about potentially life changing events, such as surgery:

**Gareth, aged 24:** *Like I love it I love the gym and I wasn’t able to go for a while… but erm, erm like my girlfriend at the time sent me all these like models who had (stoma) bags, like fitness models – and she was like it’s doable.*

Nevertheless, IBD caused significant disruption to family life for some people whereby, *‘they all had to adjust their lives around it as well’* (Jack aged 22). Those who were parents or carers described the guilt they felt when they were unable to look after their loved ones, or when their family members had put their own lives on hold to care for them. As the following quote illustrates, concerns about burdening family members during IBD flare ups meant that some did not always ask for help even when they were aware they needed support.

**Fearne, aged 38*:*** *I can’t look after my children properly – that impacts on my partner and and his job… And my mum and stuff has to help…I also feel guilty because I know I know it impacts them, so I try and I try not to ask for help even though when sometimes I may need it because I do feel l like to – I don’t feel like you know, I don’t want to be a burden on anyone.*

### Sharing experiences and reducing stigma- the benefits of social media communities

Only three participants had attended a face to face IBD support group, two of whom had attended one meeting. However, even one attendance was viewed positively, enabling shared experiences of IBD in an open and relaxed environment:

**Maddy, aged 22:** *it’s kind of being united with other people and being able to discuss it openly and have almost a laugh about it when you need to kind of thing and have it serious when you need to, so it’s just other people, a friendship I guess from it*

The main source of additional information and ongoing support post diagnosis for all participants was social media. The existence of a *“proper community”* (Sally, aged 27), online was perceived to ease the transition to life with chronic illness, whilst learning about other people’s journeys acted as a source of inspiration and a *“heads up”* (Sally, aged 27) for potential challenges in the future.

**Bobby, aged 39:** *I wish this was around when I was first diagnosed twenty years ago, because I would have been able to deal with things a lot better.*

**Stella, aged 21:** *I just like seeing other people … having so much confidence and I’m like oh I’ll be like that one day.*

Social media was described as having a positive impact on increasing awareness and reducing the stigma of IBD, particularly for the younger generation as they are *“more influenced by that”* (Abbie, aged 20).

**Katie, aged 32:** *there needs to be more knowledge out there of it but I think a lot of people online are doing it for them, I think they are doing good for exposing bags* (stoma bags).

## Discussion

The study provided insight into patient perspectives of living with inflammatory bowel disease, and to our knowledge, is one of the first to explore their access to information and support when they are adjusting to living with Crohn’s and Ulcerative Colitis. Consistent with other studies,^26^ our findings highlight the frustration that patients feel during prolonged diagnosis processes. Such frustration appeared to be exacerbated by misdiagnosis,^27,28^ leading to further suffering that negatively impacted upon quality of life. Some participants felt that general practitioners didn’t believe their symptoms, or focussed on specific symptoms whilst ignoring others.

Lack of information has been shown to result in a loss of trust in healthcare practitioners and increased feelings of fear, which can have negative implications for patient self-management of their disease.^29^ Upon IBD diagnosis, many were relieved that they were finally going to be taken seriously by medical professionals.^30^ However, despite the majority of patients having little prior knowledge of IBD, only a few received detailed information about IBD from their doctor. Our findings suggest that fear about their diagnosis may have been exacerbated by information sought online; the content of which some found overwhelming. Online information caused initial panic and anxiety, as many assumed the negative experiences they read about would happen to them. Healthcare professionals should therefore consider providing literature on IBD at the point of diagnosis to ensure patients have access to balanced, trustworthy information. Our findings suggest this should include basic information about what IBD is, as well as how it may impact day to day life depending on disease severity. Key information is available on the NHS website, including links to access additional support groups and counselling.

Periods of poor mental health, anxiety, reduced confidence, feeling a loss of control and reduced quality of life were described during periods of illness.^8,9,31,32^ Some felt guilty when IBD disrupted day to day life. Even during remission, the fear of the unknown can increase levels of stress. Mental health support for those living with IBD is a crucial, yet currently neglected element of IBD care.^33^ Our findings also highlight the importance of emotional and practical support from partners and family members. We found that patient experiences of living with IBD was not always negative.^34^ Resilience is defined as the ability to adapt from adversity, yet there is a lack of research that has explored resilience in patients living with IBD. Our findings suggest that despite facing many challenges, participants had adapted to living with IBD with a new-found confidence and determination. New knowledge, such as how to eat a healthy diet, and learning how to maintain a positive attitude, had appeared to help individuals during times of pain and illness. All participants acknowledged that although life with a chronic illness presents challenges, it also presented new opportunities facilitated by a changed perspective. This is an important finding to communicate, as knowledge of such positive experiences may offer hope to those who are newly diagnosed.

Support provided by specialists including IBD nurses and surgeons was highly valued. Indeed, participants described how such access to information and support had changed their life for the better and appeared to help regain trust in health care practitioners. Interestingly, we found that views and experiences of social media also appeared to change post diagnosis. All participants had continued to use social media, despite initial concerns, although it should be noted that the recruitment process may have influenced such findings as participants were all social media users. Social media appeared to ease the transition to life with a chronic illness, enabling connections and insight into the experiences of others living with IBD. These online communities were viewed as a source of inspiration and knowledge about potential challenges that may occur in the future.^35^ The sharing of stoma bag pictures on social media such Facebook, Twitter and Instagram were seen as having a positive impact by helping to raise awareness of IBD amongst the wider public, and potentially reduce the stigma associated with having a stoma bag. As few participants reported attending IBD support groups in person, such online communities appear to provide an important resource for individuals adjusting to life with IBD. However, social media platforms contain unregulated information, which could influence patient’s decisions about disease management.^36,37^ This finding is not specific to our sample, as previous research^38^ has underlined negative implications of patients with asthma, coronary heart disease and diabetes using the internet and social media for health information. It is therefore important that social media is not the only source of information for patients, and that doctors are aware that patients may have potential misunderstandings or misconceptions about their clinical care as a result.

### Limitations

As our sample were recruited via social media their views and experiences in relation to online information and support may not reflect patients who do not access social media. Indeed, the influence of social media may be inflated in the findings of our study as most participants were recruited via social media after they were identified as being vocal about their IBD experiences, meaning they were likely to interact with other accounts posting IBD related content. All participants were white, from the UK and accessed National Health Service (NHS) care. Our findings do not reflect the views or experiences of ethnic minority groups or those accessing support outside of the NHS. Our sample was limited to adults aged 20 – 40 years and does not reflect the experiences of older age groups living with IBD.

## Conclusion and future areas of research

Overall, our findings highlight the importance of clear communication and support from health professionals. Patients diagnosed with IBD would benefit from being given information about what IBD is, as well as how it may impact on day to day life from doctors so that social media is not the only source of information at the point of diagnosis. Participant reports suggested poor mental health and reduced quality of life during periods of ill health, and highlight the importance of professional support; particularly as some were reluctant to access care and burden family members when they were ill. Further research is needed to fully explore the extent to which parents of young children or carers do not seek medical intervention when needed, including avoiding hospitalisation, due to concerns about not being able to look after their children and burdening others. It is important that this is investigated further as medical treatment is essential to prevent flares and reduce the risk of colorectal cancer,^39^ and, if IBD is left untreated, the prognosis will likely worsen, including an increase in severe relapses and irreversible bowel damage.^40,41^ However, experiences of living with IBD were not always negative and some found new confidence and resilience, which may offer hope to those who are newly diagnosed. Social media, including anti stigma campaigns, appeared to help ease the transition to life with a chronic illness, enabling connections and insight into the experiences of others living with IBD. Further research is needed to help understanding what factors enhance or hinder resilience in people living with IBD, as well the potential role that online communities may play in facilitating protective factors associated with resilience and helping patients adjust to living with IBD.

## Data sharing

Consent was not sought from participants for data sharing.
